# Tachyarrhythmias in congenital heart disease

**DOI:** 10.3389/fcvm.2024.1395210

**Published:** 2024-06-03

**Authors:** Jaume Francisco-Pascual, Núria Mallofré Vila, Alba Santos-Ortega, Nuria Rivas-Gándara

**Affiliations:** ^1^ Unitat D'Arritmies, Servei de Cardiologia, Hospital Universitari Vall D'Hebron, Vall d’Hebron Institut de Recerca (VHIR), Vall d’Hebron Barcelona Hospital Campus, Barcelona, Spain; ^2^ Departament de Medicina, Universitat Autònoma de Barcelona, Bellaterra, Spain; ^3^ CIBER de Enfermedades Cardiovasculares (CIBER-CV), Instituto de Salud Carlos III, Madrid, Spain

**Keywords:** congenital heart disease, arrhythmias, rhythm disorders, tachycardia, sudden cardiac death

## Abstract

The prevalence of congenital heart disease (CHD) in adult patients has risen with advances in diagnostic and surgical techniques. Surgical modifications and hemodynamic changes increase the susceptibility to arrhythmias, impacting morbidity and mortality rates, with arrhythmias being the leading cause of hospitalizations and sudden deaths. Patients with CHD commonly experience both supraventricular and ventricular arrhythmias, with each CHD type associated with different arrhythmia patterns. Macroreentrant atrial tachycardias, particularly cavotricuspid isthmus-dependent flutter, are frequently reported. Ventricular arrhythmias, including monomorphic ventricular tachycardia, are prevalent, especially in patients with surgical scars. Pharmacological therapy involves antiarrhythmic and anticoagulant drugs, though data are limited with potential adverse effects. Catheter ablation is preferred, demanding meticulous procedural planning due to anatomical complexity and vascular access challenges. Combining imaging techniques with electroanatomic navigation enhances outcomes. However, risk stratification for sudden death remains challenging due to anatomical variability. This article practically reviews the most common tachyarrhythmias, treatment options, and clinical management strategies for these patients.

## Introduction

The number of adult patients with congenital heart disease (CHD) has significantly increased in recent years, primarily due to technological advances in diagnostic and surgical techniques (1). A considerable proportion of these patients are prone to developing arrhythmias because of surgical modifications, such as suture lines and prosthetic materials like patches, as well as hemodynamic alterations arising from long-term pressure or volume overload, atrial and ventricular remodeling, and, in some cases, cyanosis. Up to 50% of patients with CHD may experience supraventricular arrhythmias (SVAs), and in certain CHDs, such as Tetralogy of Fallot (ToF), ventricular arrhythmias may occur in up to 14%, carrying a non-negligible risk of SD (2).

Arrhythmias in patients with CHD have been associated with increased morbidity and mortality, exerting a negative impact on quality of life (3). Additionally, according to the Dutch Concor registry, arrhythmias constitute the most frequent cause of hospital admission in patients with CHD, accounting for up to 31% of cases (4). For instance, in a Canadian study involving over 38,000 patients with CHD, the presence of atrial arrhythmias was associated with a 50% increased risk of mortality and a 50% increase in morbidity in the form of stroke or heart failure (5). This worse prognosis may be more pronounced when considering populations with more complex cardiac conditions. For example, in palliative transposition of the great arteries (TGA) with atrial switch surgery, atrial arrhythmias increase the risk of mortality fivefold. Similarly, in patients undergoing Fontan surgeries, the risk increases sixfold (6). In all CHDs, the most common cause of sudden death (SD) is arrhythmic (7). Arrhythmias represent an increase of up to more than three times the risk of SD in the general population with CHD (8), with two potential mechanisms: complete atrioventricular block and ventricular arrhythmias.

In this article, the most common tachyarrhythmias, treatment options, and clinical management strategies for these patients are discussed. The information provided is mostly based on a narrative review of the literature, incorporating the authors' own experience in the subject as well.

## Arrhythmia substrates in congenital heart disease

Patients with CHD can be affected by various types of arrhythmias, including intranodal reentry tachycardias, accessory pathway tachycardias, focal atrial tachycardia, macroreentrant atrial tachycardias (MRAT), atrial fibrillation (AF), ventricular tachycardia (VT), and ventricular fibrillation. MRAT are the most common arrhythmias in patients with CHD. Cavotricuspid isthmus-dependent flutter (CTI), similar to the general population, remains the most frequently reported, accounting for up to 40%–60% of these arrhythmias (2, 9).

The occurrence of each type of arrhythmia is determined by the timing and type of repair, the scars generated, and the subsequent remodeling. Therefore, each form of CHD is associated with different types of arrhythmia (1, 10) (Table 1). The Fontan procedure and physiological correction of TGA (Senning and Mustard), which involve creating suture lines and altering hemodynamics, render these patients the most predisposed to developing SVAs. This risk can reach up to 50% for patients with the classic Fontan procedure at 10 years post-surgery (11). Furthermore, these patients often have poorer hemodynamic tolerance due to limited distensibility of neo-atria, dysfunction of the single ventricle or systemic ventricle, and volume or pressure overload resulting from valve abnormalities or residual shunts.

**Table 1:** Risk of arrhythmias according to congenital heart disease type.

CHD type	Atrial tachycardia	Atrial fibrillation	Ventricular tachycardia	Accessory pathways
ASD	↑↑	↑↑↑		
VSD	↑		↑	
CoA		↑	↑↑	
AV canal	↑			
Ebstein's anomaly	↑↑		↑	↑↑↑
ToF	↑↑		↑↑	
TGA (atrial switch)	↑↑↑	↑↑	↑↑	
ccTGA	↑	↑	↑	↑
LVOT obstruction			↑↑	
Classic Fontan	↑↑↑	↑		
Lateral tunnel Fontan	↑			

Summary of the risk of specific types of arrhythmias according to the type of congenital heart disease (1).

CHD, congenital heart disease; ASD, atrial septal defect; VSD, ventricular septal defect; CoA, aortic coarctation; ToF, tetralogy of Fallot; TGA, transposition of the great arteries; ccTGA, congenitally corrected transposition of the great arteries; LVOT, left ventricular outflow tract.

In other CHD, pre-existing arrhythmias may be observed prior to surgical intervention. This is evident in cases such as atrioventricular connections in patients with Ebstein's anomaly and congenitally corrected transposition of the great arteries (ccTGA).

## Management of arrhythmias in congenital heart disease

### Arrhythmias diagnosis

The diagnosis of symptomatic arrhythmias in patients with CHD is conducted similarly to the general population. Diagnostic tools such as cumulative Holter monitoring, 2-week continuous monitors, single-lead ECG devices integrated into smartwatches and smartphones, and implantable loop recorders (ILRs) are available (12–20).

In patients presenting with syncope, the ILR and electrophysiological study play an important role. In patients with a high clinical risk profile, especially those with myocardial scar or conduction disturbances, EPS not only yields a substantial number of diagnoses but also identifies patients at lower risk of arrhythmic syncope (21–29). However, its negative predictive value has been considered to be suboptimal (around 70%) (24, 30), and in such cases, further work up might be needed. The implantation of an ILR enables prolonged cardiac monitoring, offering the possibility to correlate arrhythmic events with symptoms (17, 21, 22, 31–33). This allows for additional diagnostic yield and is considered safe (17, 21, 22, 31–33).

Screening asymptomatic patients with periodic assessments beyond 12-lead ECGs or periodic Holter monitoring is of uncertain value, as a high prevalence of asymptomatic findings has been reported that seldom impact management (12, 34). Further studies are necessary to determine whether early event detection and intervention can reduce morbidity and mortality in this patient cohort.

### Hemodynamic assessment and treatment

Similarly to the general population, the initial step in the treatment of arrhythmias depends on their hemodynamic tolerance. SVAs may be poorly tolerated and can manifest as heart failure, shock, syncope or even electromechanical dissociation in extreme cases (2, 5, 21, 35–36). In the case of MRAT, the presence of patches and surgical scars leads to a slowing of conduction velocity, resulting in slower atrial cycles during tachycardia. This favors faster atrioventricular conduction, leading to hemodynamic compromise and even myocardial stunning. Patients with highly complex CHDs, such as TGA, those with dysfunction of the systemic ventricle, or severe dilation of the venous atrium, are at a higher risk of experiencing severe clinical manifestations during tachycardia episodes (37). If the patient exhibits hemodynamic compromise, urgent cardioversion should be considered. Anteroposterior pad placement is usually suitable for most patients and provides the highest rate of success (2). It should be adapted to the cardiac position (e.g., in patients with dextrocardia). In the case of SVAs, some patients, such as those with TGA, may experience sinus arrest or severe bradycardia after cardioversion, often requiring the administration of atropine/isoproterenol or external cardiac stimulation.

If patients with ASDs demonstrate good tolerance and a duration >48 h, the presence of cardiac thrombus should be ruled out through transesophageal echocardiography or appropriate anticoagulation (for more than 3 weeks), and medications for heart rate control (beta-blocker or calcium antagonist, depending on the characteristics of the heart disease) should be considered before cardioversion (1). In the case of ventricular tachycardias with good hemodynamic tolerance, the administration of intravenous drugs (amiodarone or procainamide) may be considered before considering electrical cardioversion (ECV).

In addition, reversible causes such as hyperthyroidism, an inflammatory process, or anemia need to be ruled out. In patients with CHD, the presence of hemodynamic alterations should be assessed, as they may influence the onset of arrhythmias and warrant consideration of targeted treatment. For example, in patients having undergone classic Fontan surgery, associated with a high prevalence of MRAT; in some cases, a conversion to Fontan surgeries with intra or extracardiac tunnel may be considered. Other examples include possible intracardiac shunts, progressive valvular abnormalities, etc.

**Figure 1:**
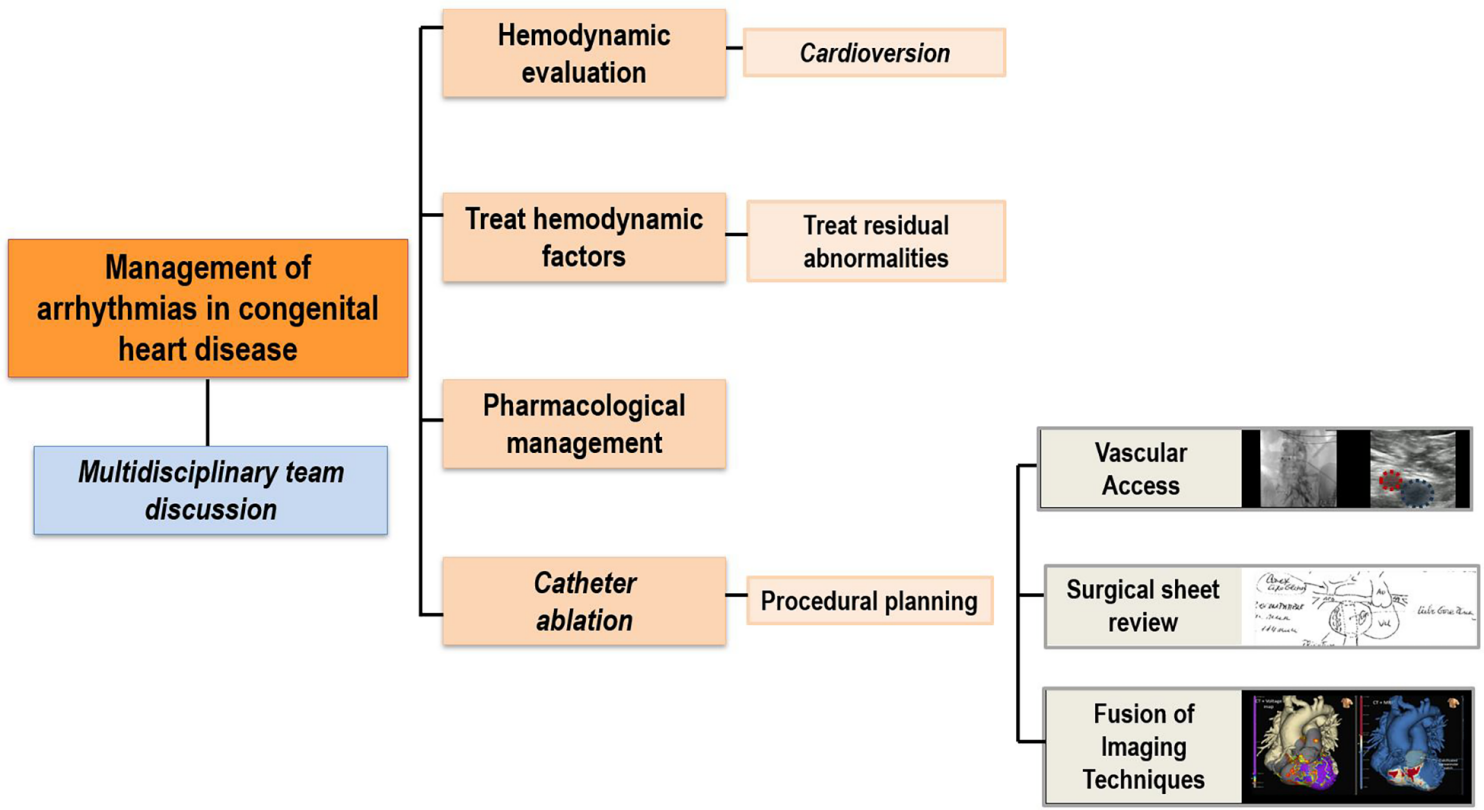
General arrhythmia workflow. Diagram outlining the description of the main steps in the evaluation and treatment of arrhythmias in patients with congenital heart diseases.

For a comprehensive assessment of all these factors, it is essential to promptly refer the patient to a specialized center with a multidisciplinary team experienced in the treatment of arrhythmias in patients with CHD (Figure 1).

### Pharmacological management

#### Anticoagulant therapy

Regarding anticoagulant drugs, there is no clear consensus on indications, and specific risk stratification is needed. Thromboembolic cerebrovascular complications are a fundamental cause of morbidity in the CHD population. According to the TACTIC study, thromboembolic events are more associated with the complexity of the heart disease than with risk scales used in the general population. However, the CHA2DS2-VASc score may be applicable in simple CHD (38). Anatomical groups with a higher known embolic risk include uncorrected cyanotic heart diseases, Eisenmenger physiology, uncorrected atrial septal defects, and Fontan circulation (2).

#### Antiarrhythmic therapy

There is a lack of data due to the heterogeneity and limited number of patients with CHD included in the studies. Rhythm control is the preferred approach in this population, as the loss of sinus rhythm is frequently poorly tolerated, especially in patients with complex cardiac conditions. Similarly to the general population, antiarrhythmic drugs have low efficacy and significant adverse effects, both extracardiac and cardiac, due to negative inotropic and/or dromotropic effects.

In the acute treatment of arrhythmias, adenosine is the drug of choice for tachycardias involving the AV node and serves as a differential diagnostic method. Special care must be taken in patients with a low ejection fraction whose cardiac output depends on heart rate. It is noteworthy that in patients with Fontan physiology, adenosine may be ineffective due to its rapid metabolism through enzymatic degradation in blood and peripheral tissues, without manifesting its cardiac effects (2).

In terms of chronic treatment, class I antiarrhythmic drugs are recommended for patients without significant ventricular dysfunction, hypertrophy, or atrial scars. However, they are not recommended for treating MRAT, as their effect on slowing intra-atrial conduction may facilitate faster ventricular responses.

Class III antiarrhythmic drugs, specifically amiodarone, are the most commonly used in these patients, particularly in those with moderate and severe CHD. Amiodarone is highly effective for maintaining sinus rhythm and treating chronic ventricular arrhythmias; however, it is associated with many long-term side effects. Up to 56% of significant side effects were reported in a cohort of patients with moderate and complex CHD during a median follow-up of 2.7 years, with 30% experiencing amiodarone-induced thyrotoxicosis, more frequently observed in Fontan physiology patients. These adverse effects led to discontinuation of the drug in 42% of cases, despite its effectiveness in those who tolerated it, with an overall or partial efficacy rate of 98% (39). Caution is advised in cyanotic patients or those with pre-existing thyroid, pulmonary, or hepatic dysfunction.

Sotalol is preferred in young patients due to its fewer side effects. As has been reported in a case series with moderate and complex CHD, sotalol has proven to be reasonably well-tolerated (only 18% discontinued treatment primarily due to fatigue or dyspnea) and safe (no arrhythmias or sudden deaths were documented, and only 13% of patients experienced significant bradycardia, mainly those with Fontan surgery). Sotalol was completely or partially effective in 94% of patients (40).

There is limited experience with dronedarone in this population, but it may be another therapeutic option that has the advantage of avoiding the thyroid effects associated with amiodarone. Liver function should also be monitored, and its use is contraindicated in heart failure (41).

Finally, beta-blockers are recommended for both heart rate control in SVAs and non-sustained ventricular arrhythmias. In acute management, they should be administered carefully to patients with ventricular dysfunction due to their negative inotropic effect. Calcium channel blockers and digoxin may also be useful for rate control.

### Catheter ablation

Due to the limitations of antiarrhythmic drugs, catheter ablation (CA) has become the first-line treatment for arrhythmias in CHD (2, 5, 42–46). This procedure represents a challenge, given the anatomical complexity and the frequent difficulty in vascular access. Meticulous procedure planning is crucial, considering the patient's anatomical characteristics and surgical techniques (2, 24, 45). Correct identification of the specific conduction system in relation to individual anatomy is essential in these patients. This is particularly important in the ablation of some arrhythmias, such as intranodal reentrant tachycardias, or in VT in patients with rToF with surgical closure of VSD, in whom the ablation site may be more challenging to locate due to variability in reference structures, increasing the risk of complete atrioventricular (AV) block.

For all these reasons, patients should be referred to centers with experience in arrhythmia ablation in CHD, especially in the case of complex CHD (47). Careful pre-procedure planning is always important.

#### Vascular access

**Figure 2:**
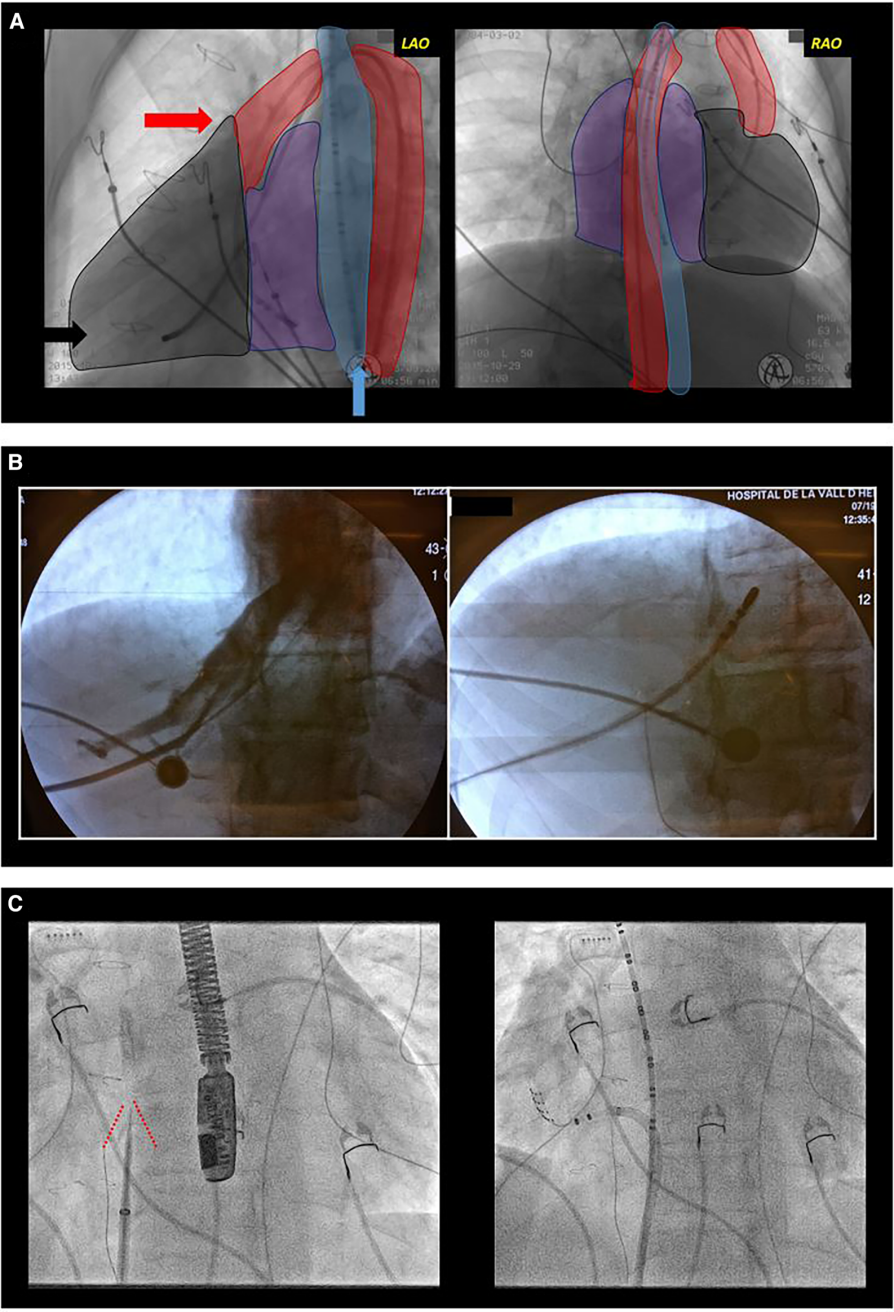
Difficult access. (**A**) Complex congenital heart disease with a single ventricle type, mesocardia, and dextroapex, initially repaired with classic Fontan surgery which was later converted to Fontan with intracardiac tube. Electrophysiological study was performed due to syncopal supraventricular tachycardia. Conventional radiological projections are shown: left anterior oblique (LAO, left image) and right anterior oblique (RAO, right image). The outlines of the esophagus (blue), aorta (red), ventricle (black), and atria (purple) are shaded. Due to the absence of femoral venous access, a transesophageal diagnostic catheter (blue arrow) was introduced for atrial sensing, and through the femoral artery, a tetrapolar catheter to the ventricle (black arrow) for rescue ventricular stimulation and a mapping-ablation catheter for treatment (red arrow) were progressed. (**B**) Transhepatic access guided by fluoroscopy. In the first fluoroscopy image, contrast injection into the hepatic venous system can be observed. In the second image, the catheter is progressed through this venous system. (**C**) Transeptal access in a patient with transposition of the great arteries corrected with atrial switch surgery (Senning). Both fluoroscopy images show an left anterior oblique projection. In the left image, a transeptal puncture needle oriented anteriorly and towards the right shoulder. Tenting of the interatrial baffle is observed (red lines). In the right image, a mapping-ablation catheter has been advanced through a long sheath to the neo-pulmonary venous atria.

In the planning of ablation procedures for patients with CHD, it is necessary to anticipate potential challenges with conventional femoral accesses. It is not uncommon for these patients to experience femoral vein occlusion, inferior vena cava atresia, or surgical obstacles such as prostheses or patches (Figure 2). To overcome these challenges, alternative vascular access, including internal jugular vein, subclavian vein, transapical, or transhepatic access may be necessary (48). In certain cases, a transesophageal catheter is employed, particularly in pediatric cases with difficulties in vascular access, to obtain intracardiac electrograms (49). Performing complex accesses, such as transeptal or transpatch procedures, requires significant electrophysiology expertise, especially in patients with corrected TGA using Senning or Mustard surgery or those with Fontan physiology involving an intracardiac tube. Utilizing intraprocedural imaging techniques like intravascular ultrasound or the fusion of previous imaging tests and electroanatomic mapping (EAM) enhances procedural guidance (50). Pre-procedural imaging tests, such as computed tomography (CT), play a crucial role in assessing vascular accesses beforehand and serving as an intraprocedural reference.

#### Anatomical knowledge

**Figure 3:**
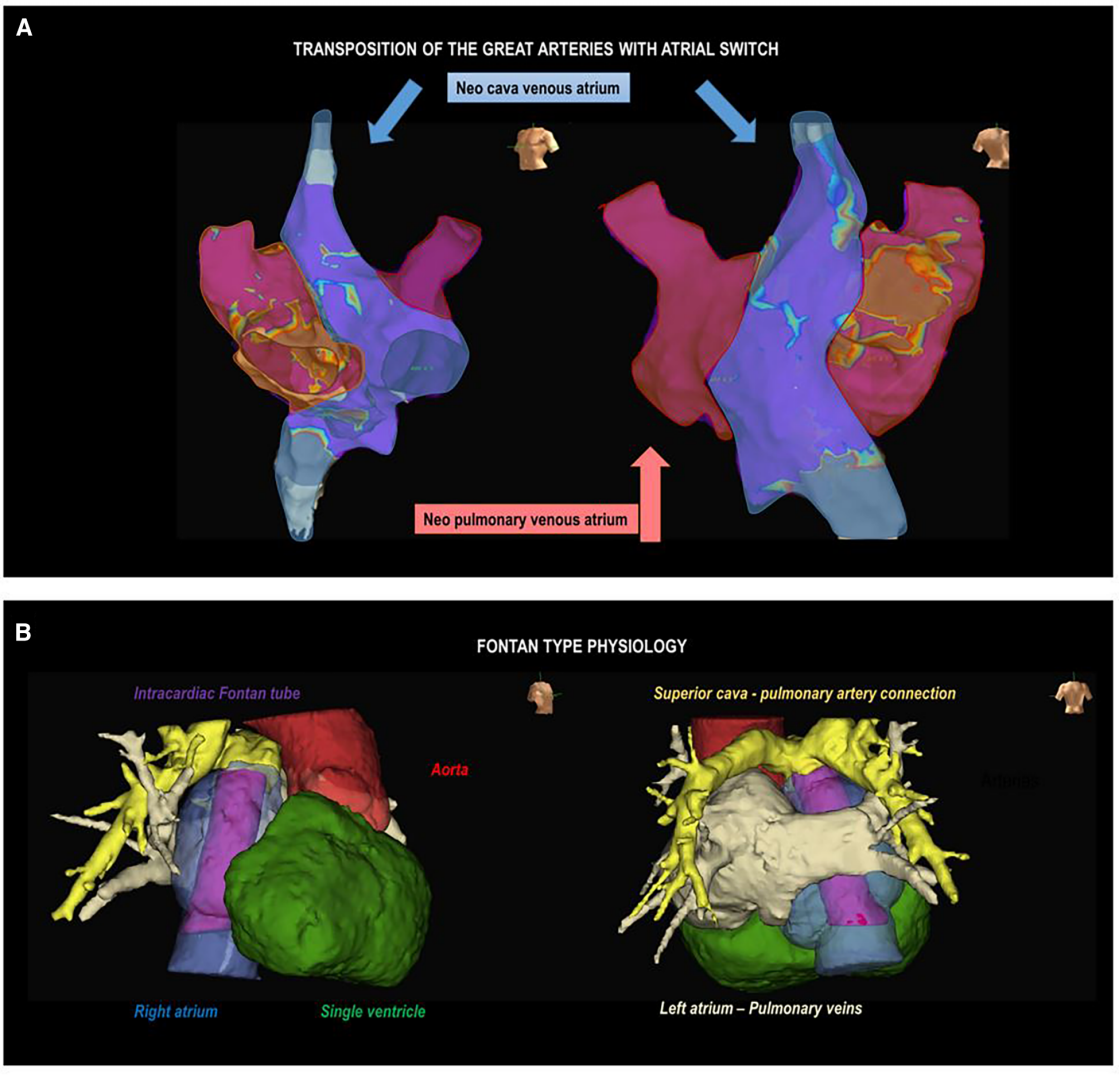
Complex anatomies. (**A**) Electroanatomic voltage map of the atrium in a patient with a transposition of the great arteries corrected with atrial switch surgery (Senning). Left oblique (left) and right posterolateral (right) projections are observed. The shaded areas represent the neopulmonary vein atrium (red) and the neocaval vein atrium (blue). (**B**) Anatomical reconstruction using image post-processing software in a patient with Fontan surgery, mesocardia, and dextroapex. A right lateral projection (on the left) shows the intracardiac tube (purple), right atrium (blue), aorta (red), and single ventricle (green); and a posteroanterior projection shows the superior vena cava-pulmonary artery connection (yellow) and left atrium-pulmonary veins (beige).

Accurate knowledge of the patient's anatomy is crucial, requiring a comprehensive examination of surgical records for those who have undergone cardiac repair. Evaluation of ventricular function and identification of residual hemodynamic anomalies, such as leaks or valve issues, is essential. These findings have implications for arrhythmias and may influence the planning of ablation procedures. The presence of significant hemodynamic abnormalities can significantly influence the occurrence of arrhythmias, prompting consideration for repair if necessary. Some of these alterations may impact ablation procedures, either by facilitating access to cardiac chambers (for example, in cases of residual communications between neo-atria in patients having undergone Senning or Mustard surgeries) or by acting as an anatomical barrier to accessing specific regions (for example, in cases of percutaneous pulmonary valve prostheses in ToF or mechanical prostheses in any location) (Figure 3).

Conventional imaging methods such as echocardiograms may be insufficient, requiring right and left heart catheterization and cardiac magnetic resonance imaging (MRI) for detailed assessments, especially in complex cases like univentricular hearts or tetralogy of Fallot. The increasing use of three-dimensional models in CHD patients enhances understanding and aids in planning interventions and educating medical professionals, patients, and families (51).

#### Fusion of imaging techniques

Multimodality imaging techniques, particularly cardiac MRI and CT, are increasingly crucial for diagnosing and monitoring CHD patients. These techniques not only aid in pre-ablation planning but also serve as intraprocedural guidance by fusing with EAM. Fusion has proven beneficial in complex heart conditions, such as VT and left-sided flutter ablation, enhancing outcomes (52–54). In patients with ToF, the correlation of cardiac MRI findings with EAM has also been demonstrated (55). Substrate characterization often involves image post-processing software (e.g., ADAS Galgo Medical), enhancing integration for comprehensive planning and guidance during procedures.

#### Ablation techniques

**Figure 4:**
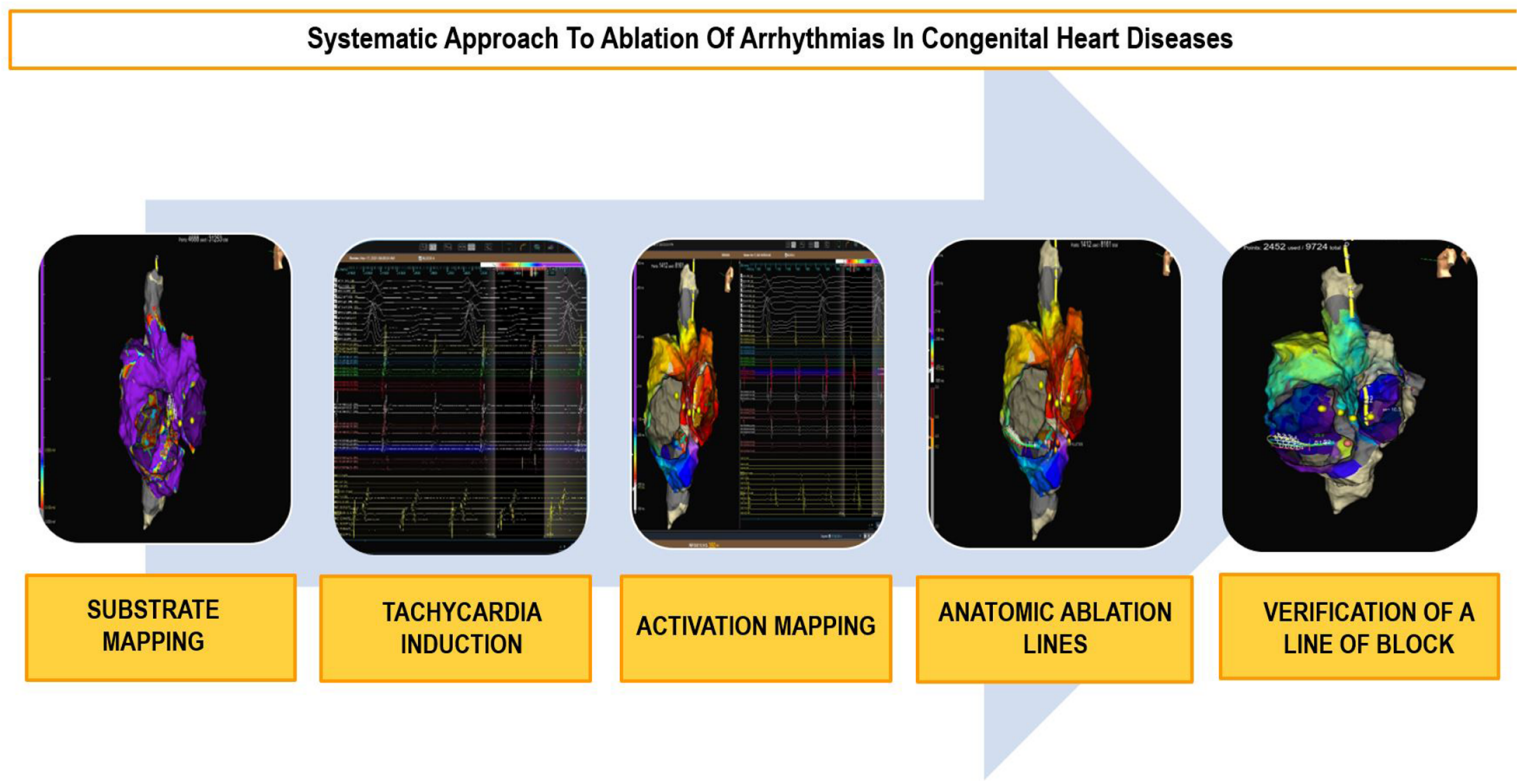
Ablation workflow in congenital heart diseases. Diagram outlining the systematic approach to ablation in congenital heart diseases in five steps.

Because of the anatomical difficulties and the high prevalence of complex arrhythmias in CHD patients, systematic planning of these procedures using electroanatomic navigation systems is recommended (2, 42, 44, 56). Continuous technological advancements in navigation systems allow for better anatomical and substrate characterization, such as identifying surgical scars and areas of fibrosis. This aids in the identification of reentries and the planning of ablation lines (Figure 4). High-density mapping catheters provide precise mapping, even in challenging accesses like transhepatic or trans-baffle, contributing to the prediction of ablation success (57, 58).

Electroanatomic navigation systems offer the advantage of reducing radiation exposure. Fusion systems, combining fluoroscopy with EAM (Carto, Univu, Biosense Webster), intravascular echocardiography with EAM (Cartosound, Biosense Webster), and transesophageal echocardiography with fluoroscopy, reduce radiation exposure and enhance mapping efficiency (59). These advances are also very useful in those patients that require a transseptal or trans-baffle approach. Image techniques are often necessary to ensure the correct localization of the needle tip before puncture and to avoid potential complications, especially in those patients with more distorted anatomies. In addition to puncture, trans-baffle access can be challenging due to tissue rigidity, which may sometimes require the use of steerable sheaths or ones with a smoother profile, or even dilation of the same.

Beyond access difficulties, the anomalous location of the specific conduction system poses a challenge in CHD patients. Cryoablation is frequently used in the ablation of arrhythmias near the AV node, offering reversible lesions and greater stability due to catheter adhesion to the endocardial surface. Its safety and moderate effectiveness were demonstrated in a series of complex CHD cases (60). For ablations in other locations, irrigated-tip catheters with steerable sheaths that facilitate the introduction of catheters into areas with complex access are recommended. Intracardiac echocardiography is acquiring an increasing role in this type of ablations. Among other benefits, it allows live visualization of the real anatomy, planning of ablation lines, and also ensures optimal tissue contact.

Remote magnetic navigation is a technological solution adaptable to CHD patients, enabling access to unconventional areas and reducing the risk of perforation. Its main limitations are its cost and availability. Its utility has been demonstrated, for example, for retroaortic access to the atrium of the pulmonary veins in cases of TGA with Senning or Mustard surgery. Remote magnetic navigation has proven to be effective and safe in this context (61).

## Specific arrhythmia types

### Atrial tachycardia

Atrial tachycardia (AT), predominantly MRAT, is the most frequently documented arrhythmia in patients with CHD. For example, in recent studies (2, 42, 62), incidences of 30% have been described in patients with Ebstein's anomaly and TGA treated with atrial switch, 20% in patients with atrial septal defects and in patients with repaired TOF, or even up to half of patients undergoing Fontan surgeries.

The natural history of AT varies significantly depending on the type of CHD. In patients with complex heart diseases, AT tends to occur at an earlier age and with a higher arrhythmic burden (62). Thus, in patients with univentricular physiology and in TGA, the onset of arrhythmias usually occurs around the second decade of life, whereas in patients with atrial septal defects, it is delayed until the fifth decade (62).

**Figure 5:**
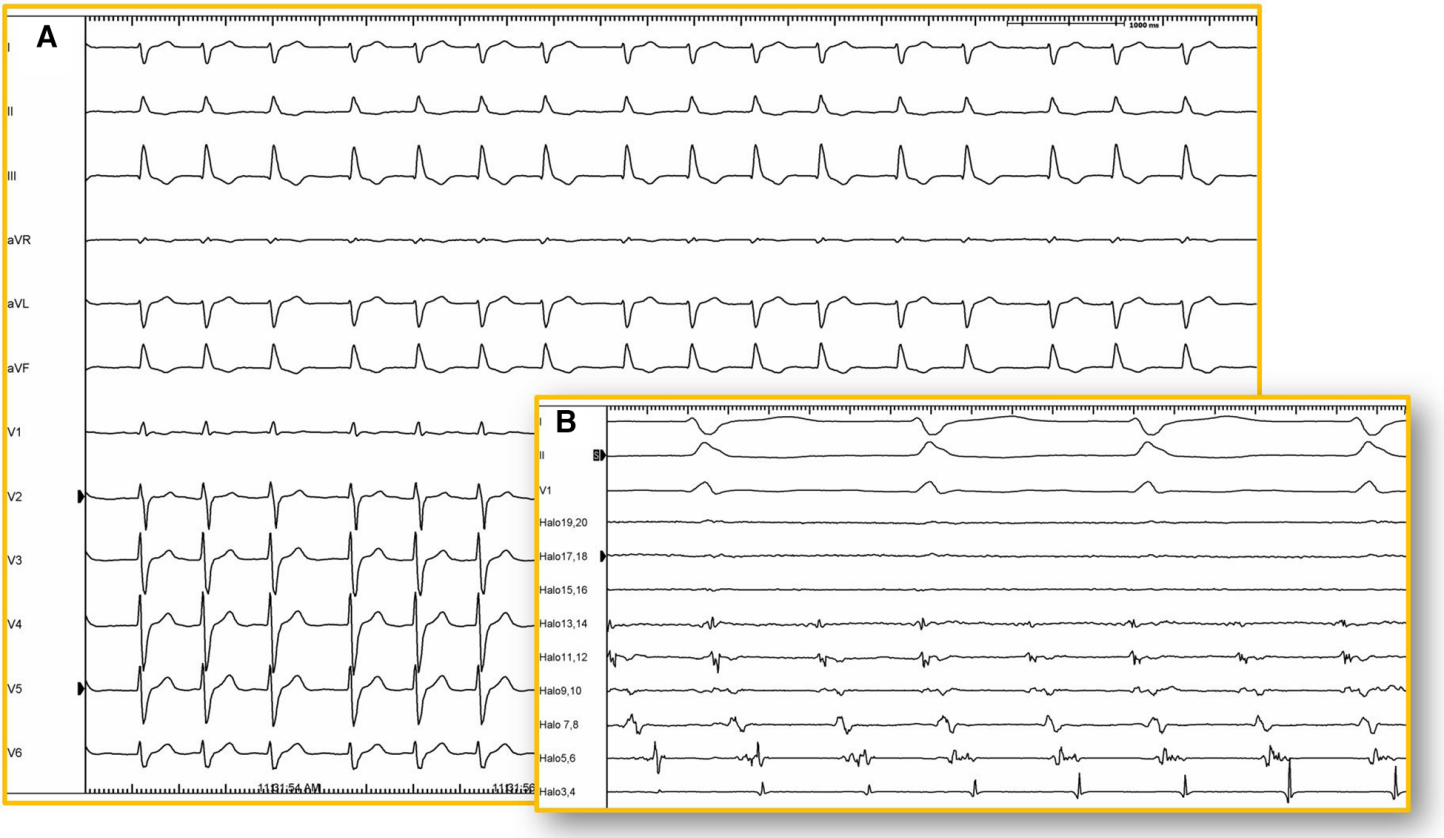
12-lead ECG. (**A**) Twelve-lead electrocardiogram (25 mm/s) of a patient with corrected d-TGA using the Senning technique. Atrial activity is very difficult to visualize due to its low amplitude and may be interpreted as atrial fibrillation. However, in the intracavitary recording (**B**) of the same patient, atrial activity is shown recorded by a duodecapolar catheter positioned between the superior and inferior vena cava (100 mm/s), revealing organized and regular activity. Electroanatomical mapping confirmed the presence of typical atrial flutter with a circuit around the tricuspid annulus (isthmus-dependent).

The presence of surgical scars, prosthetic material, anatomic obstacles, and other areas of fibrosis demarcates tissue with slowed conduction, a crucial factor for the onset of MRAT. In these areas of anomalous tissue, there is the possibility of unidirectional block, serving as the necessary substrate for the occurrence of macroreentrant arrhythmias. Due to slowed intra-atrial conduction, MRAT involving the CTI is much more frequent than in the general population and, in fact, constitutes the most frequently encountered macroreentrant circuit in this population (2, 63, 64). However, other circuits that do not involve the CTI are also common, representing, according to some case series, more than 50% or even 70% of cases in certain substrates (65). Likewise, it is not uncommon for the same patient to have both types of tachycardia (CTI-dependent and non-CTI-dependent). In a study of 94 patients, 51% presented CTI-dependent MRAT, 21% CTI-independent MRAT, and 28% both mechanisms (63). The complexity of CHD was a strong predictor of CTI-independent MRAT. The electrocardiogram (ECG) and cardiac monitoring registers in patients with complex CHD can be challenging to interpret (2, 14, 66). On the one hand, about a third of ECGs with patterns not suggestive of CTI-dependent flutter may, in fact, be CTI-dependent (63). On the other, some patients may have very low-amplitude atrial waves due to extensive atrial fibrosis, which can even make the arrhythmia go unnoticed (67, 68) (Figure 5).

Less frequently, ATs may also manifest a focal pattern (5%–10% of cases) (2, 68, 69). Electrocardiographically, they are often indistinguishable from MRAT and the diagnosis is typically established during mapping in the electrophysiology study (EPS).

#### Antiarrhythmic therapy

There are currently no randomized clinical trials available, nor are any expected, to assess the efficacy of antiarrhythmic treatments in this population. Therefore, the evidence on pharmacological management is based on a few case series and expert opinions. Both antiarrhythmic drugs and rate-controlling agents may play a role; however, rhythm control is the preferred approach.

Amiodarone is likely the most commonly used antiarrhythmic drug, both acutely to restore sinus rhythm and chronically in this context. Ibutilide, dofetilide and sotalol may also be useful for restoring sinus rhythm. To achieve rate control, beta-blockers, calcium channel blockers, and digoxin may be useful with some considerations. Digoxin is often less effective for heart rate control during exercise in young patients, especially in cases of MRAT.

#### Catheter ablation

Catheter ablation is the first-line treatment for MRAT. While acute efficacy is high at around 80%, short-term recurrence is significant (30%–50%), due to the substantial atrial myopathy present in these patients (2, 46, 64). In a series of 130 patients with CHD treated at our center, with a high percentage of complex heart disease, during a mean follow-up of 4 years, 23% experienced a recurrence of the same arrhythmia, 14% developed another left MRAT, and 8% developed AF. The efficacy of a second procedure was high, with 78% maintaining long-term sinus rhythm (46). The presence of non-ICT-dependent MRAT and the induction or prior history of AF was associated with a higher risk of recurrences.

**Figure 6:**
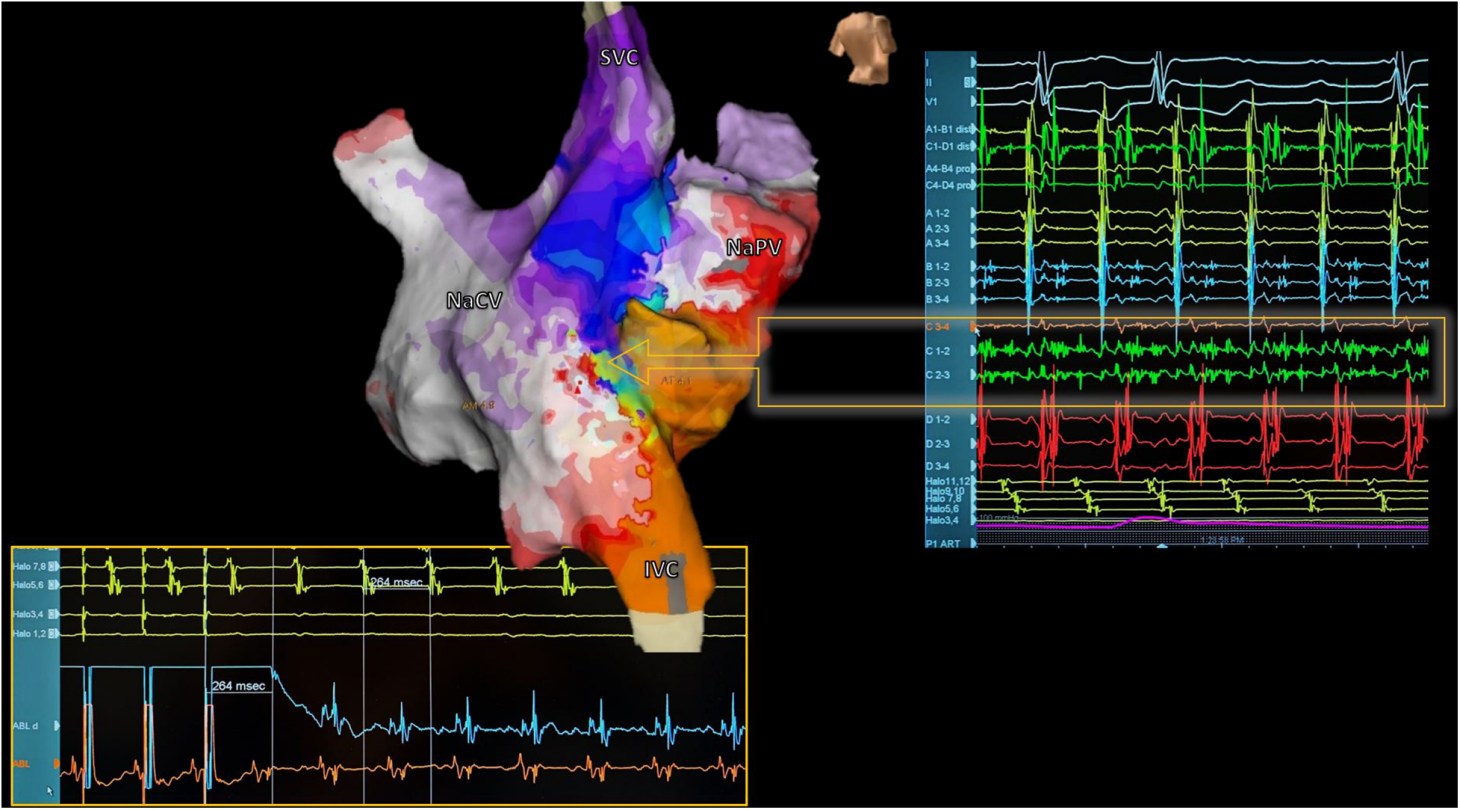
Reentrant atrial tachycardia electroanatomical mapping. Electroanatomic activation map of both neoatria during atrial tachycardia in a patient with corrected d-TGA using the Senning technique (Posterior view). Septal activation shows reentry-compatible activation (Arrow): Careful analysis of electrograms during mapping with a multipolar catheter identified a zone with fragmented and pandiastolic electrograms highly suggestive of the protected isthmus of the tachycardia (Right). At the bottom of the image, the catheter ablation maneuver positioned in this zone is shown, confirming the diagnosis. The tachycardia resolved during the first radiofrequency application. SVC, superior vena cava; IVC, inferior vena cava; NaVC, neocaval atrium; NaPV, neopulmonary vein atrium.

Meticulous procedure planning is essential, considering patient anatomical characteristics and surgical techniques. During the ablation, special attention must be paid to the location of the phrenic nerve and the conduction tissue, which may be anatomically displaced. It is highly useful to have a multipolar catheter around the AV ring in the venous atrium (e.g., a duodecapolar catheter) and in the coronary sinus. Not only does this allow for the evaluation of activation patterns, but it also aids in diagnosing changes in MRAT (Figure 6).

**Figure 7:**
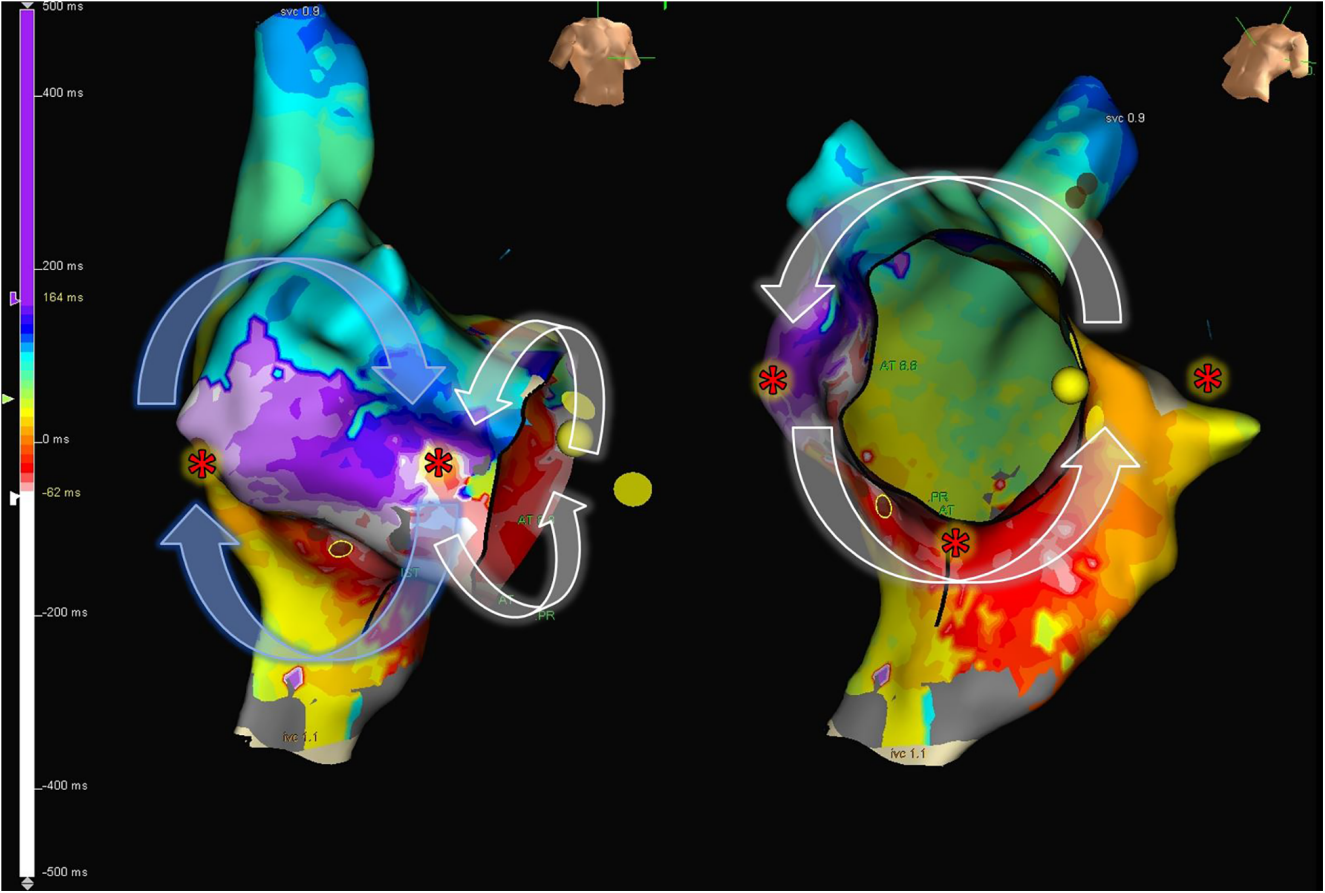
8-shape figure atrial flutter. Electroanatomic map of the right atrium, showing activation during tachycardia in a patient with repaired Tetralogy of Fallot. An 8-shaped figure with counterclockwise rotation around the cavotricuspid isthmus and clockwise rotation in the lateral wall is observed. The “*” indicates sites frequently used to perform entrainment maneuvers to determine the active circuit of the tachycardia.

Electroanatomical navigation systems are very useful when dealing with scar-related flutter, figure-eight patterns, and multiple circuits, which are more common in patients with CHD. These advanced systems enable precise and efficient characterization of chamber anatomy, identifying blocking zones and fibrosis areas essential for planning the ablation line. Despite the increasing accuracy of automatic annotation systems, traditional electrophysiology remains valuable. Operators analyzing electrograms ensure proper annotation, particularly in areas with double potentials, delayed potentials, or absence of capture, contributing to precise anatomical substrate characterization. Accurate identification of blocking zones and dense scar, and also additional diagnostic maneuvers, such as entrainment, are useful when planning the ablation line. For instance, in patients with atriotomy scars (such as those performed in the majority of repaired ToF cases), a possible figure-eight pattern may be identified after EAM, with a counterclockwise circuit in the CTI and a clockwise circuit in the lateral wall, peri-scar (63). While only a small proportion genuinely exhibit a figure-eight flutter, ablation in the CTI is often sufficient to eliminate the tachycardia. Entrainments are also important for diagnosis. A long return cycle from the posterolateral aspect of the right atrium (posterior to the atriotomy scar) will suggest a passive rotation (Figure 7).

The main objective of ablation is the termination and non-reinduction of clinical arrhythmia (2, 45, 66, 70–71). However, it is not uncommon for the patient to present in sinus rhythm, or for more than one arrhythmia to be induced. In the first scenario, it is important to conduct an atrial induction protocol (since in most cases, at least one AT can be induced) and map it. In situations where mapping and determining the critical isthmus of the tachycardia cannot be achieved, either due to a lack of inducibility or the presence of unstable arrhythmias, empirical ablation of the CTI or its equivalent would be appropriate, given that it is the most frequently involved anatomical substrate. If other arrhythmias that can be mapped are induced after the ablation of the clinical arrhythmia, these can also be ablated. Recent research indicates a lower recurrence of arrhythmias in patients who underwent ablation for all induced ATs compared to those where only the clinical arrhythmia was addressed (45). There remains some debate surrounding the need to empirically address possible anatomical isthmuses that have not been confirmed to be involved in the genesis of a clinically or induced AT.

An ablation line must be created between two anatomical obstacles or scars to interrupt the circuit, preferably addressing the protected isthmus. It is essential to emphasize the importance of verifying bidirectional block of the ablation line to prevent future recurrences. Although it may be more challenging in complex anatomies, maneuvers such as differential pacing, preferably associated with new EAM, generally allow for confirmation of the blockade of the line or for the identification of possible gaps in the event of persistent conduction.

### Atrial fibrillation

Atrial fibrillation was previously considered much less frequent than MRAT. Nevertheless, its prevalence has significantly increased in current cohorts due to improved survival of patients with complex cardiopathies (2, 62). AF often appears in patients with a history of prior MRAT, indicating a more advanced stage of atrial cardiomyopathy. A rhythm control strategy is usually preferred, as the loss of atrial contraction can be poorly tolerated. However, treatment effectiveness is suboptimal, and despite therapeutic efforts, patients may experience recurrences and a tendency toward chronicity. The results of AF ablation in patients with complex CHD have been disappointing thus far, likely because the automatic activity of the pulmonary veins (typically addressed in conventional AF ablation) represents only a small part of its pathophysiology (44). AF appears in more advanced stages of the disease, with highly fibrotic and diseased atria. Therefore, treating MRAT, which often precedes AF, in its early stages is crucial to attempting to prevent the progression of atrial cardiomyopathy secondary to the tachyarrhythmia. In this context, it is also important to assess and treat potential reversible causes, such as residual communications or obstructions, in order to reduce recurrence. Another important aspect to consider is the embolic risk and the need for anticoagulation. Commonly used scales (such as CHA2DS2-VASc) have not been validated in this population, so the decision for long-term anticoagulation should be made on an individualized basis. In patients with complex CHD with persistent or recurrent AF, long-term anticoagulation is likely indicated regardless of other thrombotic risk factors (2).

### Other supraventricular arrhythmias

Supraventricular arrhythmias, such as atrioventricular nodal reentrant tachycardia and accessory pathway-mediated tachycardia, can also occur in patients with CHD. Accessory pathways may be present in up to 20% of patients with Ebstein's anomaly. “Atypical” pathways, such as atriofascicular (Mahaim) pathways or the presence of multiple pathways, are also more prevalent in these patients (2). A nearly specific type of SVA in cases of right isomerism syndrome and congenitally corrected TGA is twin atrioventricular nodal reentrant tachycardia. This rare type of reentry occurs in patients who have two AV nodes, resulting in an atrioventricular reentry circuit between them.

Similar to the general population, CA is usually the treatment of choice, although the procedure is often more complex due to anatomical peculiarities. Nevertheless, experienced centers achieve high success rates and safety in CA. Knowledge of anatomy is crucial when planning the ablation. Special attention should be given to the location of the conduction system, as some substrates such as TGA, heterotaxy syndromes, or patients with septal defects may be displaced, increasing the risk of AV block during the procedure.

### Ventricular arrhythmias

Ventricular arrhythmias in this population include monomorphic VT, polymorphic VT, and ventricular fibrillation. Monomorphic VT is more prevalent in patients with incisions or patches in the ventricular myocardium. In patients with CHD, monomorphic VTs can be very fast and hemodynamically poorly tolerated, potentially leading to SD, even in patients with preserved ventricular function. In fact, in patients with repaired ToF and TGA who have implantable cardioverter-defibrillators (ICD), most appropriate therapies correspond to episodes of high-frequency monomorphic VT (72, 73).

#### Risk stratification

The identification of patients at a higher risk of ventricular arrhythmias would allow for the implementation of primary prevention measures and the minimization of complications from unnecessary therapies. However, the stratification of arrhythmic risk in the CHD population is complex due to the significant anatomical variability and variations in surgical repairs, the low SD rate, and the need for prolonged follow-up, making randomized clinical trials challenging. Although moderate-to-severe ventricular dysfunction, especially of the systemic ventricle, is a risk factor associated with SD, patients with preserved or only slightly impaired ventricular function may experience fatal arrhythmic events. Repaired ToF (rToF) is the best-studied cardiac condition, with multiple risk factors associated with ventricular arrhythmias and/or SD having been described (Table 2) (74–77).

**Table 2:** Predictors of ventricular arrhythmias and sudden death in repaired tetralogy.

Group	Risk factor	Author/year
Demographic	Age	Katz. 1982
MR late enhancement	Myocardial fibrosis	Babu-Narayan. 2006
Surgical	Age at corrective surgery	Gatzoulis. 2000
	Palliative surgery	Khairy. 2008
	Ventriculotomy	Khairy. 2008
Electrocardiographic and Holter	High-grade ventricular premature beat	Harrison. 1997
	Non-sustained VT	Harrison. 1997, Khairy. 2008
	Sustained atrial arrhythmias	Valente. 2014
	QRS >180ms	Gatzoulis. 1995
	QT dispersion >70%	Gatzoulis. 1997
	QRS fragmentation	Bokma. 2016
Hemodynamic	Moderate-severe RV systolic dysfunction	Knauth. 2006, Valente. 2014
	Severe RV dilation	Knauth. 2006
	Disproportionate RV hypertrophy	Valente. 2014
	Moderate-severe LV systolic dysfunction	Ghai. 2022, Knauth. 2006, Valente. 2014
	Severe pulmonary regurgitation	Ghai. 2022
	Elevated LV telediastolic pressure	Khairy. 2008
Electrophysiological study/EAM	Induction of sustained VT	Khairy. 2004
	Isthmus of slow conduction	Kapel. 2016
	Prolonged RV activation time	Rivas-Gándara. 2021
	HV interval >55 ms	Rivas-Gándara. 2021

EAM, electroanatomic mapping; MR, magnetic resonance; VT, tachycardia ventricular; RV, right ventricle; LV, left ventricle.

In patients with rToF, the occurrence of non-sustained ventricular tachycardia identifies a specific subset of patients who require careful monitoring. Some accessible non-invasive markers, such as filtered QRS duration, low amplitude signal duration in the terminal portion of the filtered QRS complex and the ratio of the maximum short-axis diameters of the right and left ventricles have been associated with potentially malignant ventricular arrhythmias (78, 79).

However, when considered individually, the predictive value of these risk factors is limited. Therefore, the indication for implantation of an ICD in primary prevention in patients with repaired ToF, aside from severe dysfunction of the systemic ventricle (left ventricular ejection fraction <35%) in patients in NYHA functional class II-III (class IIa), is based on the combination of several risk factors (class IIa), including systolic or diastolic dysfunction of the left ventricle, documentation of non-sustained VT, QRS duration ≥180 ms, inducibility of sustained VT in EPS, and the presence of extensive fibrosis in the right ventricle analyzed by cardiac magnetic resonance imaging (1, 55, 80).

In recent years, new tools for stratifying the risk of SCD in patients with CHD have emerged, such as the PREVENTION-ACHD risk score model (81). This model incorporates common clinical variables like coronary artery disease, NYHA class II/III, supraventricular tachycardia, systemic ejection fraction <40%, subpulmonary ejection fraction <40%, and QRS duration. Its accuracy exceeds current guideline indications, potentially aiding in the selection of CHD patients who may benefit from preventive implantable ICD implantation (81).

#### Mechanism and anatomic substrate of ventricular tachycardia

**Figure 8:**
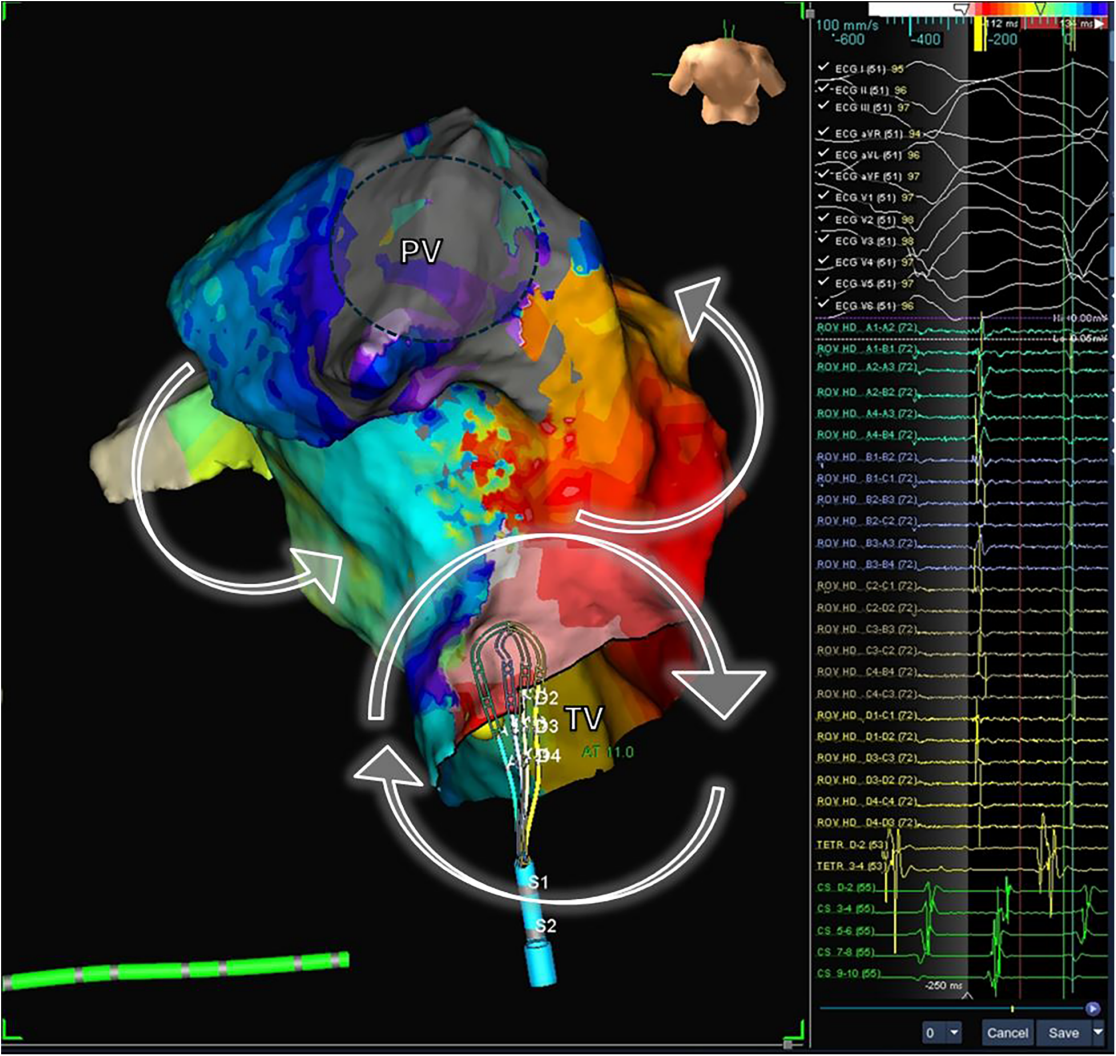
Electrophysiological study and electroanatomic mapping of ventricular tachycardia. The electroanatomic activation map of a ventricular tachycardia in a patient with repaired Tetralogy of Fallot is shown. The mechanism of VT is a macro-reentrant circuit with clockwise activation around the tricuspid valve (VP) and counterclock activation around the pulmonary valve (PV). The mapping catheter is located in the critical isthmus, showing mid-diastolic potentials (right panel). PV, pulmonary valve; TV, tricuspid valve.

Patients with CHD exhibit a substrate that predisposes them to ventricular arrhythmias, due to a combination of surgical scars, anatomical barriers, and myocardial tissue injury. Repaired ToF is the most emblematic example of CHD repair with this substrate. Studies have demonstrated that the most common ventricular arrhythmia in patients with repaired ToF is monomorphic ventricular tachycardia (82), and the underlying mechanism is macro-reentry (83). These macro-reentrant circuits are typically situated in the right ventricular outflow tract (RVOT) and are associated with the repair surgery (83, 84). Previously, ToF repair surgery involved a ventricular approach, aiming for extensive release of the RVOT, working from the assumption that residual pulmonary insufficiency had no long-term repercussions. Consequently, in addition to the patch for closing the VSD, most patients have a ventriculotomy scar, and in many cases, a transannular patch for RVOT enlargement (Figure 8). The substrate formed by surgical repair and myocardial changes due to hemodynamic overload from residual lesions, with residual pulmonary insufficiency being the most common, is responsible for the development of ventricular arrhythmias in adulthood among these patients.

#### Electroanatomic mapping

**Figure 9:**
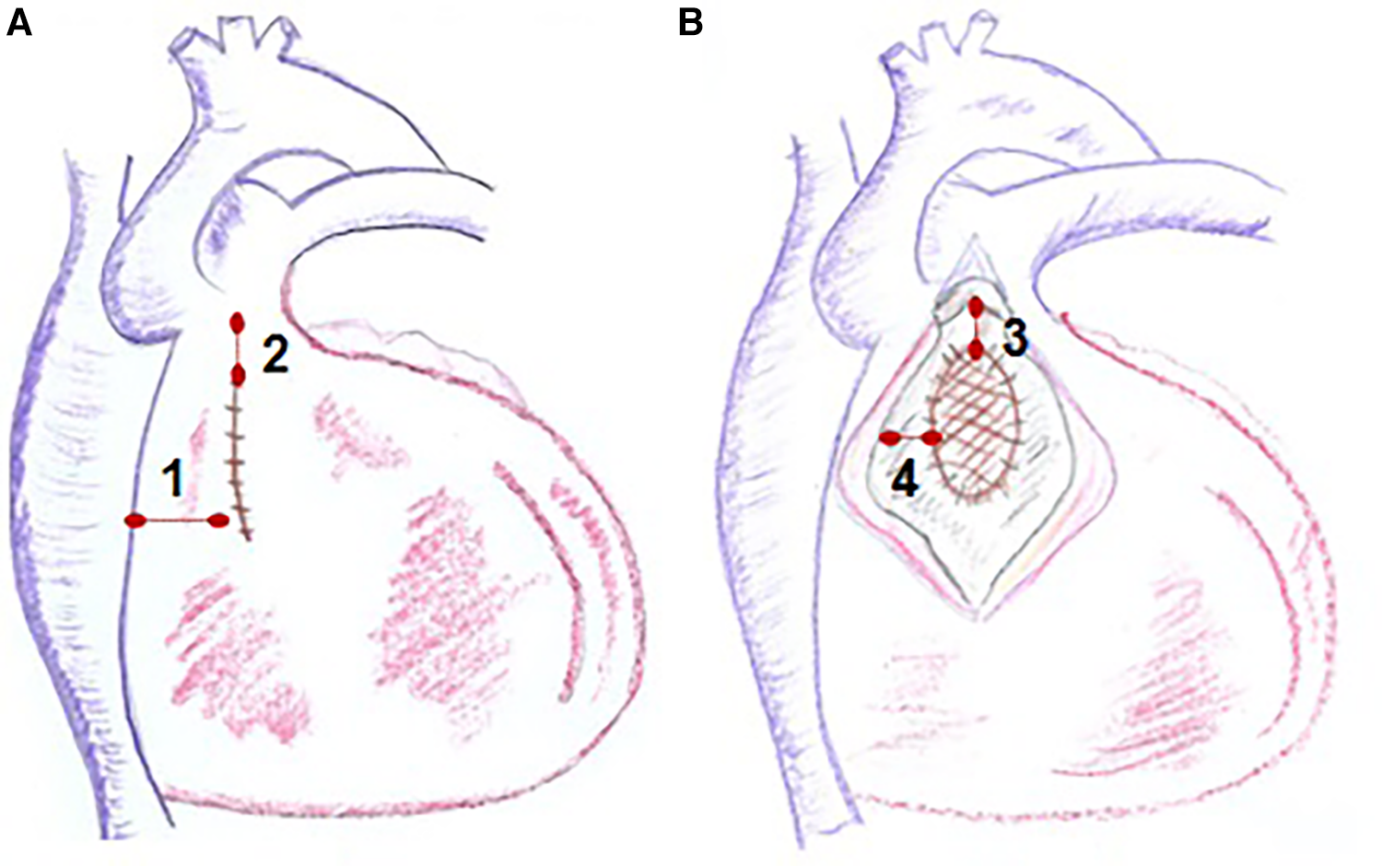
Anatomical isthmuses in repaired tetralogy of fallot. Diagram of the anatomical isthmuses responsible for ventricular tachycardia (VT) in repaired ToF, as defined by Zeppenfeld et al. (**A**) Between ventriculotomy or transannular patch and tricuspid annulus (isthmus 1), between ventriculotomy and pulmonary annulus (isthmus 2). (**B**) Between VSD patch and pulmonary annulus (isthmus 3) and between VSD and tricuspid annulus (isthmus 4). VSD, ventricular septal defect.

Electroanatomic mapping techniques, combined with electrophysiological entrainment maneuvers, enable precise identification of the macro-reentrant electrical circuit in these arrhythmias and establish the critical isthmus maintaining the arrhythmia (Figure 8). In repaired Tetralogy of Fallot (rToF), the critical anatomical isthmuses sustaining these macro-reentrant circuits are delineated by the non-excitable tissue of the valvular rings, prosthetic patches, and surgical scars. Electro-anatomic mapping of right ventricular voltage in sinus rhythm (considering electrograms >1.5 mV as high voltage and electrically non-excitable tissue as electrograms with voltage <0.5 mV and without ventricular capture with 10 mA stimulation and 2 ms) has identified four anatomical isthmuses that may be present in these patients (85). These four isthmuses are defined between the following structures: isthmus 1 between the transannular patch/ventriculotomy and the tricuspid ring, isthmus 2 between ventriculotomy and the pulmonary ring, isthmus 3 between VSD patch and the pulmonary ring, and isthmus 4 between the VSD patch and the tricuspid ring (Figure 9) (85). It is noteworthy that although the majority of rToF patients have at least one of these anatomical isthmuses, not all can propagate sustained monomorphic VT. In this regard, it has been observed that arrhythmogenic isthmuses are longer, narrower, and have a slower conduction velocity, which can help define the anatomical location of the ablation (86).

In other anatomical substrates, scientific evidence regarding the mechanism of VT and the arrhythmic substrate is limited, and additional anatomical isthmuses have been described in patients with TGA, VSD, or repaired pulmonary stenosis (87).

#### Ablation

Radiofrequency ablation of VT in patients with repaired CHD is feasible, with reported acute efficacy ranging from 50% to 100% and variable recurrence rates (85, 88). Comparing published studies is complex due to small sample sizes, the use of diverse mapping and ablation technologies, and different efficacy criteria. Kapel et al. reported an acute efficacy of 74% with no recurrences in a mean follow-up of 46 months, utilizing EAM systems and irrigated ablation catheters. The procedure was considered effective when achieving conduction block at the critical anatomical isthmus level and demonstrating absence of inducibility. Therefore, considering the macro-reentrant mechanism of these arrhythmias, it seems reasonable to aim for bidirectional block of the critical isthmus and absence of inducibility in ablation procedures.

In those patients in whom sustained monomorphic VT is induced with good hemodynamic tolerance, the identification of arrhythmogenic isthmuses for ablation can be based on electro-anatomic mapping during tachycardia and entrainment maneuvers. However, complete mapping during VT is often not possible due to lack of inducibility or poor hemodynamic tolerance (72, 73). In the case of patients with rToF, considering the association between the substrate identified in sinus rhythm and the induced VTs, substrate ablation can be considered with the goal of blocking conduction through the arrhythmogenic isthmuses considered (86).

The efficacy of ablation may be limited due to dilation of the cavities, hypertrophy of the myocardial tissue, or the presence of prosthetic material [e.g., after pulmonary valve replacement (PVR)]. In patients with rToF where blockade of septal isthmuses cannot be achieved, either due to hypertrophy or the presence of a pulmonary valve prosthesis, it may be useful to complement the ablation line contralaterally using a left-sided approach.

The role of empirical ablation at the time of PVR in patients with rToF and severe residual pulmonary insufficiency is a subject of controversy. Since residual hemodynamic defects are associated with a higher risk of VT and SD, addressing their treatment is important. However, there is doubt as to whether PVR reduces the long-term arrhythmic risk and whether it should be associated with ablation of anatomical isthmuses (89, 90). Sometimes, the arrhythmogenic substrate does not completely revert after PVR, even with overall remodeling of the right ventricle (reduction in volumes and improvement in systolic function). Additionally, access to isthmus 3 (between the pulmonary ring and VSD) may be limited after PVR due to the interposition of the prosthesis in the pulmonary position. Therefore, it seems reasonable to consider associating ablation of arrhythmogenic isthmuses before or during PVR with the aim of preventing monomorphic VTs during follow-up. Although cryoablation during PVR surgery has not been reported to be proarrhythmic and could have a protective role in the long term (59), long-term studies are needed to clarify this point.

#### Assessment of arrhythmic substrate using imaging techniques

Late gadolinium enhancement study in cardiac MRI allows the extent and distribution of fibrotic tissue to be determined. In fact, the 3D reconstruction of images obtained with late gadolinium enhancement MRI has proven to be useful for characterizing the arrhythmic substrate of the left ventricle in patients with ischemic heart disease. A good correlation has been documented in the identification of fibrosis and conduction channels between EAM and late gadolinium enhancement MRI, with the latter providing appropriate guidance for planning and guiding the ablation procedure (52, 91). However, the analysis of the right ventricle, which is often involved in VTs in patients with CHD, is more complex due to its thinner wall and the proximity of epicardial fat. There appears to be an association between the extent of fibrosis in late gadolinium enhancement MRI and the history of arrhythmias in patients with repaired Tetralogy of Fallot (92). Additionally, the 3D reconstruction of late gadolinium enhancement MRI of the right ventricle has demonstrated correlation with the substrate identified by voltage mapping, both in the extent and location of fibrosis (55, 93), as well as in the identification of anatomical isthmuses (55), making it potentially useful for guiding the ablation procedure.

#### Future perspectives

The shift in the surgical approach during corrective procedures, avoiding ventriculotomy through transatrial and transpulmonary methods, and the minimization of residual hemodynamic lesions, will modify the arrhythmic substrate of the ventricular myocardium in patients with repaired CHD. This change has the potential to influence the risk of arrhythmias and the treatment approach through ablation. The characterization of the arrhythmogenic substrate and anatomical isthmuses, achieved via 3D reconstruction of late gadolinium-enhanced MRI, EPS and EAM in sinus rhythm, could emerge as a valuable tool for risk stratification concerning VT and SD in individuals with repaired CHD (23, 55, 94, 95). Further studies are necessary to elucidate the role of empirical ablation during pulmonary valve replacement in patients with repaired Tetralogy of Fallot (96, 97).
